# Profile of Producers and Production of Dry-Aged Beef in Brazil

**DOI:** 10.3390/foods10102447

**Published:** 2021-10-14

**Authors:** Jonatã Henrique Rezende-de-Souza, Flavio Andre Bolini Cardello, Ana Paula Moraes de Paula, Felipe A. Ribeiro, Chris R. Calkins, Sérgio Bertelli Pflanzer

**Affiliations:** 1 Department of Food Engineering and Technology, University of Campinas, Rua Monteiro Lobato, 80, Campinas 13083-862, SP, Brazil; jonatarezendesouza@gmail.com (J.H.R.-d.-S.); cardello1349@gmail.com (F.A.B.C.); anamoraes.unicamp@gmail.com (A.P.M.d.P.); 2 Department of Animal Science, University of Nebraska, Lincoln, NE 68583-0908, USA; felipegea@hotmail.com (F.A.R.); ccalkins1@unl.edu (C.R.C.)

**Keywords:** Brazilian livestock, aging, beef, value addition

## Abstract

No information is currently available on the profile of producers and production process of dry-aged beef in Brazil, to the best of the authors’ knowledge. We surveyed 37 Brazilian companies that were producing dry-aged beef in 2020 to investigate this market. The absolute and relative frequency of responses was calculated to obtain the sum, average, minimum, and maximum values. From the respondents, dry-aged beef was first produced in 2009, and most producers are located in big cities. Most respondents control and monitor chamber temperature; however, humidity and air velocity only are monitored. The aging period (mostly between 22 to 60 days) was the main indicator of product readiness. The process losses (water loss and crust trimming) can reach 65%. Some producers perform microbiological analyses to ensure product safety and others use tools such as GMP and SOP. The results of this survey may help governmental institutions to develop a standardized industrial protocol for producing dry-aged beef in Brazil.

## 1. Introduction

Brazil has one of the largest livestock populations worldwide, with the world’s second largest cattle herd (~244 million head of cattle). Brazil is the second largest producer (10.1 million tons) and the largest exporter (2.6 million tons) of beef [1]. Despite the robust numbers, roughly 80% of the Brazilian herd is comprised of Zebu cattle, raised on pasture and slaughtered at an advanced age, which may result in less tender meat [2,3].

Palatability is usually associated with the perception and acceptance of tenderness, flavor and juiciness of meat. For many years, tenderness was described as the most important characteristic. Since the tenderness was improved over time, flavor started to be indicated as the first trait, followed by tenderness and juiciness. However, this may not be true of all production systems around the world [4]. Currently, the aging process is the main practice used by the industry and the retail sector to improve tenderness and is accomplished by storing the meat under refrigeration for different periods (days, weeks, or months).

Until the mid-1960s, meat was stored and distributed without any type of packaging, leading to moisture loss and characterizing the dry aging process. The emergence of plastic packaging for vacuum packaging allowed storage for longer periods and distributing meat more safely, characterizing the wet aging process [5]. Nevertheless, the dry aging process has been gaining popularity more recently, as the meat produced is characterized by an intense and desired flavor by the most consumers, despite higher cost and lower yields in the process.

The high demand for special meats in Brazil has directed enterprises, such as slaughterhouses, meat shops, or restaurants, to produce dry-aged beef, despite the lack of specific legislation for its production and marketing.

Several factors directly affect meat quality and process yield of dry-aged beef, involving raw materials, such as animal age, sex, weight, breed, amount of subcutaneous and intramuscular fat, cuts (muscles) as well as environmental factors, such as temperature, air humidity, and ventilation [6].

This work investigated the profiles of producers and the production processes of dry-aged beef in Brazil.

## 2. Materials and Methods

This research was approved by the Ethics Committee of the University of Campinas (Protocol Number: 08899319.5.0000.5404) and all volunteers expressed their consent.

Initially, we screened dry-aged beef producers in Brazil. We contacted slaughterhouses, meat distributors, and consultants via telephone and e-mail, in a standardized manner, to identify the enterprises producing dry-aged beef in the country and 37 companies participated in the survey.

The survey was conducted through an online questionnaire, applied on the Google Forms interface between August and November 2020. The questionnaire consisted of 42 questions categorized into 9 sessions, namely: general information (*n* = 3); infrastructure and production capacity (*n* = 5); origin and information on raw material (*n* = 8); process parameters (*n* = 8); business management (*n* = 4); economic issues (*n* = 3); product safety (*n* = 7); product sensory attributes (*n* = 2); and final considerations (*n* = 2).

All data were tabulated in an Excel spreadsheet (Microsoft, Redmond, WA, USA). The absolute and relative frequency of responses were calculated to obtain the sum, average, minimum, and maximum values.

## 3. Results and Discussion

### 3.1. General Information

Respondents were asked about the year when they started dry-aged beef production (Table 1). Our survey showed that the first record of dry-aged beef production occurred in 2009 by one of the enterprises. Since then, roughly one more companies started production each year until 2013. From 2014 to 2018, three to five more companies each year started producing dry-aged beef. The largest number of new companies (10) occurred in 2019, showing a sharp market increase for this product in Brazil. However, the number of new companies dropped to four in 2020, possibly due to the global health situation imposed by the coronavirus COVID-19 pandemic.

The gradual increase in the number of new producers until 2019 may be attributed to gains in the wage income of Brazilian families. According to the Brazilian Institute of Geography and Statistics (IBGE) [7], per capita income in Brazil rose from BRL 630.25 in 2009 to BRL1380.00 in 2020, an increase of 118.96% during this period. In addition, the meat market in Brazil has been growing steadily over the years, with an increase of the number of new specialized meat shops mainly in major urban centers in the country.

The southeast concentrates most dry-aged beef producers (74.4%) in Brazil, followed by the south (15.4%), and central west (5.1%), and northeast (5, 1%) (Table 1). The southeastern region houses the bigger part of the Brazilian population (42% of the Brazilian population) and has the highest average income per capita, with a value of BRL 2645.00. The northeastern region, however, has the lowest per capita income (BRL 1510.00) and the central-western region is the least populated (7.5% of the population) [7,8]. São Paulo state (southeast) has the largest number of dry-aged beef producers, which may be related to a high per capita income, in addition to its gastronomic culture. Furthermore, the capital city of São Paulo (São Paulo state) is the largest metropolis in Latin America and considered the sixth largest in the world, with over 22 million inhabitants in 2020, accounting for 50.04% of the entire population of São Paulo State [8].

Dry-aged beef in Brazil is mainly commercialized in butcher’s shops, where the meat goes through the trimming process to be sold to the consumer (40.6%). Twenty-five percent of the enterprises surveyed are restaurants, where beef undergoes culinary preparation and is served to the consumer as a ready meal in the same place of production. Sensory and convenience issues can justify this tendency because beef has a dark color and dry texture after the dry-aging process and shows a more desirable appearance with the removal of the crust surface. In addition, trimming and steak cutting for commercialization promote greater comfort and ease for consumers, associated to consumption at restaurants, as it is a ready-to-cook or ready-to-eat product.

### 3.2. Infrastructure and Production Capacity

The companies interviewed have one to five aging chambers; the majority has only one chamber (67.7%), and one of the most prevalent productions has a capacity that ranges from 100 to 1000 kg of meat (43.2%) (Table 2). Nevertheless, the total meat used for the dry-aging process increased considerably between 2017 and 2019 (Table 3).

### 3.3. Origin and Raw Material Information

Dry aging is used to enhance meat tenderness and flavor [9,10]. To meet this improvement on sensory quality, how the producers select the meat cuts (raw material) can impact the process and the final quality of the product. Table 4 shows the meat traits used by producers in dry aging. The largest part of the interviewees (48.1%) reported that the raw material received was unvacuumed in plastic packaging; 26.9% received the meat vacuum-packed, and another 25.0% received unpacked meat. For those receiving vacuum-packed meat, the combination of wet and dry aging methods can be a good strategy to reduce losses in processing and keep the desired traits of the product Meat received in plastic bags implies a reduction in the contamination risk (chemical, physical, or microbiological).

Regarding the time from the slaughterhouse to start the aging, 67.4% of the producers reported a period shorter than 7 days. The action of proteolytic enzymes in the breakdown of myofibrillar proteins begins right after animal slaughter and continues throughout the rigor mortis period and during meat storage in a refrigeration system [11,12,13]. Thus, the longer the time between slaughter and the beginning of dry aging, the more intense the action of these proteases. Conversely, this time can be critical for the sanitary product quality, as most meat is stored before transport and is transported from the packing plant to the new establishment without vacuum or even without controlled conditions of humidity and ventilation.

**Figure 1:**
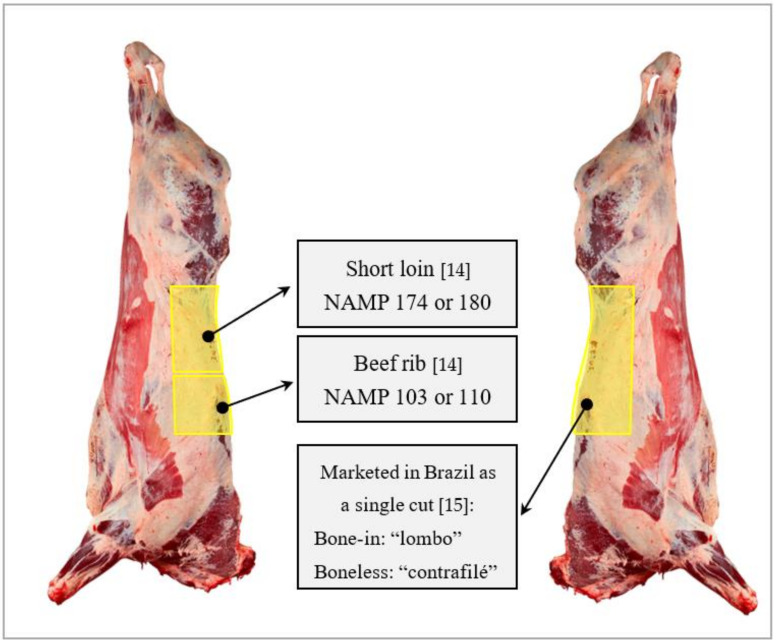
Schematic figure showing the main beef cuts used for dry aging in Brazil [14,15].

The main cuts (75.5%) used for dry aging originate from the thoracic–lumbar region, represented by beef rib (NAMP 103) and short loin (NAMP 174), which can be marketed in Brazil as a single bone-in cut with tenderloin (NAMP 103 plus 174—from the 6° thoracic vertebra to 6° lumbar vertebra) or boneless and without the tenderloin (NAMP 180—boneless strip loin plus NAMP 110—boneless beef rib) [14,15] (Figure 1). The cuts in the dorsal–lumbar region are the principal valued on the carcass and the main muscle, the Longissimus lumborum et thoracis responds well to the aging process [16]. The homogeneous fat cover, cut size and shape, and the presence of bones may explain the preference for this cut for the dry aging process.

Regarding the presence or absence of bone, 86.1% reported aging the cuts exclusively with bone. Conversely, 13.9% of the interviewees reported aging the meat with and without bone and the chuck and rump cap were mentioned among the aged boneless cuts. Bernardo et al. [17] reported that dry-aged bone-in meat has better yield and higher moisture content, without affecting the product final quality compared to dry-aged boneless meat.

In addition to bone presence, most respondents (78.4%) had requirements regarding fat cover of the meat cuts with a minimum of 5 mm reported for 48.6% of the interviewees. For the others (29.7%), fat cover should be 10 mm minimum. Bernardo et al. [17] evaluated dry-aged meats with and without subcutaneous fat and found that fat-aged meats showed better yield without affecting the quality attributes. Regarding intramuscular fat (marbling), 63.2% of the interviewees do not have specific requirement for this quality parameter for the raw material selection. The other 36.8% of respondents mentioned the existence of an individual standardization but did not provide many details. The search for marbling content standardization may be mostly attributed to the consumers’ sensory experience. According to the USDA quality rating, meat palatability can be predicted, and marbling is responsible for meat tenderness and juiciness during consumption, promoting greater lubrication during mastication [18].

Genetic is another important factor related to the raw material. In Brazil, the main genetic species used in animal husbandry is Zebu [2,3]. These animals have high calpastatin activity, an enzyme that inhibits the calpain action [19,20]. Calpains are the most important proteolytic enzymes in the aging process and consequent improvement in the sensory parameters of the meat [21]. Therefore, reduced calpain activity affects the final quality of meat.

Most dry-aged beef producers prefer meat from animals originating from a cross between Bos taurus and Bos indicus genetics or from animals exclusive of Bos taurus genetics (59.5%). Only one producer reported using meat exclusively from Zebu animals. The higher the concentration of Bos taurus genetics, the lower the calpastatin activity and consequently higher meat tenderness [19,20]. Regarding raw material requirements, 64.9% of producers reported considering weight and size of the meat cuts to be aged, and in general, respondents prefer large cuts for a better yield.

### 3.4. Process Parameters

Temperature, air humidity, and air speed are environmental factors that can affect dry aging. These factors contribute to water evaporation and concentration of flavors and aromas in dry-aged meats and are important for microbiological stability [22,23].

According to Dransfield [21], proteolytic enzymes in meat have greater activity at higher temperatures, similar to the activity in the body of a living animal. However, higher temperatures compromise the microbiological safety of meat. Therefore, a temperature close to that of melting ice in the aging process is desirable to hinder microbial development. Low temperatures favor food safety and reduce possible sensory changes due to the action of psychrotrophic bacteria. According to Feiner [24], this group of bacteria at concentrations above 5 log CFU (Colony Forming Unit) generate compounds that affect meat flavor, causing sensory rejection by the consumer. In this study, most interviewees (78.4%) control the temperature in the aging chamber. Some producers (48.6%) keep the temperature at 2–4 °C, while 29.7% of the interviewees control the temperature from −1 to 1 °C. Conversely, 21.6% of the interviewees reported that they do not have a system for controlling or monitoring the temperature in the chamber (Table 5).

Seventy-three percent of interviewees control air relative humidity. Air humidity ranges between 70% and 80% for 45.9% of the respondents, followed by 60–70% for 16.2% of producers. Values above 80% and below 60% were cited by 10.8% of respondents. Similar to room temperature, some producers (27%) reported that they do not have a system for controlling or monitoring relative air humidity in the chamber (Table 5). According to Dashdorj et al. [6], air humidity below 70% intensifies meat water loss to the external environment, reducing the process yield; nevertheless, this humidity level slows down microrganismal development. Conversely, Bernardo et al. [25], stated that relative humidity above 80% favors microbial development and contributes to surface slime growth, negative conditions for the product. In addition, producers (33.3%) reported that air humidity is mostly controlled by regulating compressors and evaporators, followed by the use of hygroscopic salts (21.4%), and humidification and dehumidifying systems (14.3%). The other producers (28.6%) only monitor air humidity, without a specific control system.

Regarding the airflow inside the chambers, most producers (67.6%) use additional fans and only one fan is used in 37.8% of the chambers. Conversely, 32.4% of respondents reported that they do not use fans. Producers were asked whether they consider air speed low, medium, or high (assessed empirically). For 56.8% of respondents, air speed in the aging chambers is medium, while the other respondents reported low and high speeds in an equivalent way (Table 4). According to Dashdorj et al. [6] and Lee et al. [22], high air flow intensifies water loss, reducing chances of microbial development. In addition, it decreases the process yield, causing the low air flow to be generate the opposite action, that is, it favors microbial development and also the process yield. Therefore, a medium air flow can result in positive process conditions, both in terms of safety and efficiency. Several techniques can be used to increase air flow in the aging chamber. Other procedures are also recommended, such as placing meat cuts to age on perforated grids and/or shelves, spacing meat cuts to allow air passage, and suspending meat cuts by hooks.

To determine the end of the dry aging process and thus market the product, 77.1% of producers reported that they mostly compute the time in days. Evaporation losses (16.7%) and customer demand (6.3%) were also mentioned as determinants for the process completion (Table 5). The most common aging times described by the interviewees range from 7 to 8 weeks (28.8%), 4 and 5 weeks (25.8%), and 6 weeks (19.7%). Some producers also mentioned aging times shorter than one week (1.5%) and longer than 8 weeks (15.2%). Dransfield [12,21] and Puga et al. [26] demonstrated that proteolysis, mainly by calpains, significantly affect the sensory perception of meat tenderization and needs to occur for at least 7 days. These authors reported that the longer the aging period, the better the meat tenderness.

Respondents reported that the decision for these process parameters were based mainly on scientific literature (37.1%), followed by trial and error (34.3%). Decisions on the process parameters are diverse due to the lack of legislation for the production of dry-aged beef in Brazil.

### 3.5. Management of the Production Process

Table 6 presents the data on the production process management. The interviewees stated that most production of dry-aged beef occurs through a continuous system (57.9%) in which new meat cuts are placed in a chamber when there are still cuts in the aging process. The other interviewees work in the batch system, where a new aging stage begins only after the previous one is complete. The frequency that new meat cuts start the aging process occurs every 30 days for most respondents (52.6%), followed by 14 (28.9%), and 7 days (18.4%). Most respondents dry age by positioning the cuts with the fat cover facing upward or sideward (77.5%). Other place forms were mentioned, such as hanging (17.5%) and fat cover facing downward (2.5%).

For protection and covering of meat cuts during aging, 24.3% of the producers use some kind of cover, either milk butter or swine lard. This aging type, popularly known as “fat cover aging”, has become common in meat stores and boutiques in Brazil. However, some producers consider it different from dry aging, mainly because when the meat is coated with the lipid layer, the water content evaporated during aging is different than when the meat is aged without covering. There is a specific packaging system on the market for the dry aging process, known as a special bag [25,27]. However, none of the interviewees acknowledged the use of this new packaging system.

### 3.6. Economic Issues

Table 7 shows data on yield and value added. Respondents (27.5%) stated that dry-aged beef loses from 11% to 15% of weight during aging (evaporation). Some producers (26.3%) reported losses greater that 25% due to trimmings. Altogether, losses can reach almost 65%. These losses are common in dry-aged meat, mainly because it is unpackaged food, which leads to water evaporation in the aging chamber [6,28]. In addition, differences in temperature, air humidity, and air speed rates, as well as the cut type and meat traits, significantly affect losses.

Regarding the value added to the product after aging and trimming, the highest proportion of respondents (45.9%) acknowledge an increase in sale values between 81 and 120% when compared to vacuum-aged meat. This value is added to the product sales price to support production costs. Studies were carried out with panelists to identify the purchase intent of dry-aged meats and, according to Smith et al. [29], 37.7% of respondents were willing pay USD 1.10 more for 0.5 kg of dry-aged beef. In turn, Berger et al. [30], demonstrated that 42.5% of respondents would be willing to invest USD 1.00 more for 0.45 kg of dry-aged beef, while 32.5% and 19. 2% would pay US@ 2.00 and US $ 3.00 more, respectively, for 0.45 kg of dry-aged beef. The data in our study and those on consumers’ purchase intent [29,30] indicate the willingness to purchase dry-aged meat, despite the higher price compared to vacuum-aged meat, possibly due to sensory characteristics, which confers to dry-aged beef a potential in this market niche.

### 3.7. Product Safety

Some respondents (27.0%) reported microbiological problems, such as mold, slime, and undesirable odors, when they started the dry-aging process in their plants (Table 8). Laboratory analyses were conducted to understand the microbial development and 45.9% of the respondents have already carried out these analyses to detect *Salmonella*, quantify total mesophiles, *Escherichia coli*, molds and yeasts, lactic acid bacteria, and thermotolerant coliforms.

The producers mentioned certain control tools to ensure product safety, such as the empirical control of the process (53.2%) with control of aging chamber conditions (temperature, air humidity, and air speed). The interviewees (32.4%) reported cleaning the aging chamber after completion of the process, while 27.0% and 24.3% of the respondents carried out the cleaning every 7 or 30 days. For 48.6% of the interviewees, the chamber is cleaned without removing the cuts, while 40.5% of the producers remove the meat cuts before cleaning the chambers, replacing them afterward. Sanitization reduces the number of microbial cells in the environment; however, when it is carried out with meat cuts still inside the aging chamber, meat cuts may be contaminated by chemical contaminants (cleaning products) and physical contaminants (dust and old meat pieces).

For 97.3% of respondents, beef is dry-aged in exclusive chambers, while some producers (2.7%) use the chambers for dry and vacuum-aged meat. The use of the chamber to age packaged meats or other products can result in cross-contamination, compromising product and consumer safety. Most producers (78.4%) reported having a specific area with specific equipment and utensils for handling aged meat, in addition to being an air-conditioned environment.

### 3.8. Sensory Attributes

Table 9 presents data on sensory aspects. Producers reported that the predominant flavor descriptor in their meats were buttery flavor (37.7%), intense meat flavor (21.3%), and almond flavor (18.0%). The interviewees also mentioned the most striking sensory characteristics of products from market competitors. The main traits regarded the meat positive characteristics (53.7%), such as tenderness, slight sweet presence, intense umami taste, and the presence of intense meat, buttery, almond, and cheese flavors. The strange aspects cited by 22.0% of the respondents referred to the accentuated presence of acid, bitter taste, and flavors related to rancid and putrid, as well as the presence of surface slime and mold in the meats. The classification of “others” expressed the lowest number of responses (11.5%), which refers to meats with no flavor or meats with sensory quality that was not different in flavor to those sold by the respondent. The main positive flavors reported by the producers are also most cited by the researchers who conduct sensory evaluations of dry-aged beef [28]. According to Lee et al. [22], these flavors are formed due to the process of proteolysis and lipolysis, generating peptides and free fatty acids, respectively. Setyabrata et al. [31] reported that other metabolites are generated and are all responsible for the characteristic flavor of dry-aged meats.

### 3.9. Final Remarks

In this survey, we asked producers about their main challenges for dry-aged meat production. We computed 10 attributes, highlighting the absence of parameters for process standardization (27.3%) and national legislation on dry-aged meats and consequent lack of inspection by responsible public bodies (20.5%) (Table 10).

To date, in Brazil, the Non-Objection Term is the only official information provided by the Ministry of Agriculture, Livestock and Supply, with administrative process no. 21000.001987 of 2018 [32]. This term provides a list of basic technical traits as suggestions of conditions for the production of dry-aged meat, and it applies only to meat industries, not to the retail sector, which is inspected by the Health Surveillance Agency (ANVISA). Nevertheless, as it is only a non-objection term and it comprises a suggestion for the production process, Brazilian producers rely solely on technical and scientific literature, which does not provide process standardization.

The relevant information of the production process, mentioned by producers of dry-aged meats, shows eight attributes and the most cited were: the product is a delicacy (22.2%), and the process must be respected (22.2%) and regulated (16.7%) (Table 10).

## 4. Conclusions

The present survey provides an overview of producers and process profiles of dry-aged beef currently applied in Brazil. Generally, we can conclude that the dry-aged beef market has been expanding in Brazil. Most producers are located in large urban centers and have low-volume productions, selling their products directly to consumers at meat boutiques or restaurants. Producers are concerned with the standardization of raw materials, requesting whenever possible, meat from heavy animals, with known genetics, and with good fat cover on the meat. The main aged cuts are from the lumbar region, bone-in, and aged for 3 to 8 weeks.

Chambers condition, such as temperature and relative air humidity, varies in a large range. The air humidity is not controlled but is monitored, and the most cited range is from 70% to 80%, while 2 to 4 °C is the most frequent range cited for temperature. Losses by evaporation and trimming reported by respondents are variable, but they are consistent with the literature, as well as valuation of the dry-aged beef at the time of sale to the consumer. Thereby, this study can aid in the development of protocols for new producers or help create specific norms by government institutions to provide a standardized industrial protocol to dry-aged beef producers.

## Figures and Tables

**Table 1:** General identification of enterprise that produce dry-aged beef in Brazil.

Item	Answers	
	N°	%
When did you start producing dry-aged beef?		
2009	1	2.7
2010	1	2.7
2011	1	2.7
2012	0	0.0
2013	1	2.7
2014	3	8.1
2015	3	8.1
2016	4	10.8
2017	4	10.8
2018	5	13.5
2019	10	27.0
2020	4	10.8
Total	37	100
Where is your company located (State/Region)?		
Bahia—Northeast Region	1	2.6
Espírito Santo—Southeast Region	1	2.6
Mato Grosso—Central-West Region	1	2.6
Mato Grosso do Sul—Central-West Region	1	2.6
Minas Gerais—Southeast Region	2	5.1
Paraná—South Region	2	5.1
Rio de Janeiro—Southeast Region	1	2.6
Rio Grande do Norte—Northeast Region	1	2.6
Santa Catarina—South Region	4	10.3
São Paulo—Southeast Region	25	64.1
Total	39	100
How do you market dry-aged beef?		
Trimmed meat for culinary preparation by the consumer	26	40.6
Untrimmed meat for culinary preparation by the consumer	8	12.5
Culinary preparation in the commercial establishment itself	16	25.0
Hamburger production	2	3.1
Sale to catering companies	1	1.6
Sale to other companies that market the meat	11	17.2
Total	64	100

**Table 2:** Production capacity of dry aging producers in Brazil in 2019.

Item	Answers	
	N°	%
How many aging chambers does your company have?		
1	25	67.6
2	8	21.6
3	2	5.4
4	1	2.7
5	1	2.7
Total	37	100
What is the production capacity of dry-aged beef?		
<100 kg	15	40.5
101 to 1000 kg	16	43.2
>1000 kg	6	16.2
Total	37	100

**Table 3:** Total, minimum and maximum values of dry-aged beef production in Brazil *.

Year	Total (kg)	Minimum (kg)	Maximum (kg)
2017	47.675	5	30.000
2018	88.470	20	60.000
2019	181.330	20	100.000

* 2020 data were not presented by all producers and are therefore not described in the table.

**Table 4:** Information on raw materials used for dry-aged meat in Brazil.

Item	Answers	
	N°	%
How do you receive the raw material?		
Packed with vacuum-free plastic packaging	14	26.9
Packed with vacuum plastic packaging	25	48.1
Unpacked	13	25.0
Total	52	100
What is the duration time between slaughter and receipt of the raw material?		
Less than 7 days	29	67.4
Between 8 to 14 days	13	30.2
Longer than 21 days	1	2.3
Total	43	100
What meat cuts do you dry-age?		
Chuck	5	10.2
Eye of rump	1	2.0
Rib and Loin cuts	37	75.5
Rump cap	1	2.0
Carcass quarters	5	10.2
Total	49	100
Is the meat aged boned or boneless?		
Exclusively boned	31	86.1
Boned and boneless	5	13.9
Exclusively boneless	0	0.0
Total	36	100
Do you have any requirement for meat fat cover?		
Yes		
Greater than 5 mm	18	48.6
Greater than 10 mm	11	29.7
No	8	21.6
Total	37	100
Do you have any requirements regarding the meat marbling degree?		
Yes	21	56.8
No	16	43.2
Total	37	100
Do you have any requirements regarding animal genetics?		
Yes		
Pure bred or crossed *Bos taurus*	22	59.5
*Bos indicus*	1	2.7
No	14	37.8
Total	37	100
Do you have any requirements regarding the cut weight and size?		
Yes	24	64.9
No	13	35.1
Total	37	100

**Table 5:** Conditions of dry-aged meat production process in Brazil.

Item	Answers	
	N°	%
What is temperature in the aging chamber?		
Exclusively from +2 to + 4°C	18	48.6
Exclusively from −1 to + 1°C	11	29.7
Does not have a thermometer	8	21.6
Total	37	100
What is humidity in the aging chamber?		
Less than 60%	2	5.4
60 to 70%	6	16.2
70 to 80%	17	45.9
Higher than 80%	2	5.4
Does not have control	10	27.0
Total	37	100
How do you control the air relative humidity in the aging chamber?		
Water	1	2.4
Hygroscopic salts	9	21.4
Compressors and evaporators	14	33.3
Humidifier/Dehumidifier	6	14.3
Does not have a specific system	12	28.6
Total	42	100
How many fans do you have in the aging chamber?		
Natural chamber ventilation	12	32.4
One fan	14	37.8
Two fans	4	10.8
Three or more fans	7	18.9
Total	37	100
What is the air speed in the aging chamber?		
High	8	21.6
Medium	21	56.8
Low	8	21.6
Total	37	100
What criteria do you use to determine the process completion?		
Time in days	37	77.1
Weight loss	8	16.7
Demand	3	6.3
Total	48	100
How long is the meat dry-aged?		
Less than 8 days	1	1.5
From 8 to 15 days	3	4.5
From 15 to 21 days	3	4.5
From 22 to 35 days	17	25.8
From 36 to 42 days	13	19.7
From 43 to 60 days	19	28.8
More than 60 days	10	15.2
Total	66	100
How did you define the process parameters you currently use?		
Scientific literature	26	37.1
Trial and error	24	34.3
Other professionals	20	28.6
Total	70	100

**Table 6:** Information on the management of the dry-aged meat production process in Brazil.

Item (Sum of Frequency of the Items Surveyed)	Answers	
	N°	%
Do you use the batch or continuous system?		
Continuous	22	57.9
Batch	16	42.1
Total	38	100
How often do the chambers receive more fresh meat?		
Weekly	7	18.4
Fortnightly	11	28.9
Monthly	20	52.6
Total	38	100
How do you position the meat cut in the chamber?		
Fat cover up or to the side	31	77.5
Fat cover down	1	2.5
Hanging	7	17.5
Does not control the meat cut position	1	2.5
Total	40	100
Do you use any packaging or cover in the meat for dry aging?		
No	28	75.7
Yes, fat cover	9	24.3
Total	37	100

**Table 7:** Economic issues related to the production of dry-aged meats in Brazil.

Item	Answers	
	N°	%
What is the average evaporative loss after aging?		
<10%	4	10.0
11 to 15%	11	27.5
16 to 20%	9	22.5
21 to 25%	9	22.5
>25%	6	15.0
Did not respond	1	2.5
Total	40	100
What is the average loss for trimming after aging?		
<10%	4	10.5
11 to 15%	7	18.4
16 to 20%	8	21.1
21 to 25%	8	21.1
>25%	10	26.3
Did not respond	1	2.6
Total	38	100
What is aggregate relative price in relation to vacuum-aged meat?		
21 to 40%	1	2.7
41 to 60%	4	10.8
61 to 80%	3	8.1
81 to 100%	9	24.3
101 to 120%	8	21.6
>121%	6	16.2
Did not respond	6	16.2
Total	37	100

**Table 8:** Safety of dry-aged meat production in Brazil.

Item	Answers	
	N°	%
Did you ever have a problem with microbiological contamination?		
No	25	67.6
Yes	10	27.0
Other initial problems	2	5.4
Total	37	100
Have you ever carried out microbiological analyses of aged meats?		
No	17	45.9
Yes	13	35.1
No, but intends to	7	18.9
Total	37	100
What actions do you take to ensure product safety?		
Empirical evaluation of the process, with adjustments of humidity and temperature, when necessary	25	53.2
Care with quality tools (GMP * and SOP **) and chamber hygienization	12	25.5
Frequent microbiological and physical chemical analyses	10	21.3
Total	47	100
What is the cleaning frequency of the aging chamber?		
Periodically	2	5.4
At the end of each process	12	32.4
Weekly	10	27.0
Fortnightly	3	8.1
Monthly	9	24.3
Quarterly	1	2.7
Total	37	100
Do you remove the meat cuts before cleaning the chamber?		
No	18	48.6
Yes	15	40.5
Cleaning between changes of batches	4	10.8
Total	37	100
Is your chamber exclusive for dry aging?		
Yes	36	97.3
No	1	2.7
Total	37	100
Does your company have a specific area for handling dry-aged meat?		
Yes	29	78.4
No	8	21.6
Total	37	100

* Good Manufacturing Practice; ** Standard Operating Procedures.

**Table 9:** Sensory attributes of dry-aged meat in Brazil.

Item	Answers	
	N°	%
Describe the flavor of your dry-aged meat:		
Buttery	23	37.7
Almond	11	18.0
Cheese	7	11.5
Intense meat flavor	13	21.3
Others	7	11.5
Total	61	100
What draws the attention of competitors’ dry-aged meats?		
Strange aroma and flavor aspects	9	22.0
General appearance, aroma, and flavors aspects	6	14.6
Positive aroma and flavor aspects	22	53.7
Others	4	9.8
Total	41	100

**Table 10:** Final considerations of dry-aged meat producers in Brazil.

Item	Answers	
	N°	%
What were or are the main challenges to produce dry-aged meats?		
Competition	1	2.3
Consumer knowledge	4	9.1
Cost of quality assurance analysis	1	2.3
Equipment	4	9.1
Financial	2	4.5
Legislation and inspection	9	20.5
Process standardization	12	27.3
Quality of raw material	1	2.3
Refrigeration	1	2.3
No challenges	9	20.5
Total	44	100
Describe any relevant information about the process or product:		
Make consumers aware of the product	2	11.1
Have good partners	2	11.1
Regulation	3	16.7
It is a delicacy	4	22.2
The process must be respected	4	22.2
The process is expensive	1	5.6
The process is new	1	5.6
The process is simple	1	5.6
Total	18	100

## Data Availability

The data presented in this study are available in the article.
